# CT and MR imaging of orbital inflammation

**DOI:** 10.1007/s00234-018-2103-4

**Published:** 2018-10-11

**Authors:** Teresa A. Ferreira, P. Saraiva, S. W. Genders, M. V. Buchem, G. P. M. Luyten, J-W Beenakker

**Affiliations:** 1 Department of Radiology, Leiden University Medical Center, Albinusdreef 2, 2333 ZA Leiden, The Netherlands; 2 Department of Radiology, Hospital da Luz, Estrada Nacional 10, km 37, 2900-722 Setubal, Portugal; 3 Department of Ophthalmology, Leiden University Medical Center, Albinusdreef 2, 2333 ZA Leiden, The Netherlands; 4 Department of Radiology, C.J.Gorter Center for High-field MRI, Leiden University Medical Center, Albinusdreef 2, 2333 ZA Leiden, The Netherlands

**Keywords:** Orbital inflammation, Orbital inflammatory diseases, CT, MRI, Diffusion-weighted imaging

## Abstract

**Purpose:**

Orbital inflammation can be idiopathic or in the context of a specific disease and it can involve different anatomical orbital structures. On imaging, inflammatory disease is frequently mistaken for infection and malignant tumors, and its underlying cause is often not determined. Through this article we aim to improve orbital inflammation diagnosis and underlying inflammatory diseases recognition.

**Methods:**

The imaging protocols and characteristics of orbital inflammation were reviewed.

**Results:**

A decision tree for the evaluation of these patients is provided. First, a combination of clinical and radiological clues is used to recognize inflammation, in particular to differentiate it both from orbital infection and tumor. Subsequently, different radiological patterns are recognized, often allowing the differentiation of the several orbital inflammatory diseases.

**Conclusion:**

The use of adequate imaging protocols and subsequent evaluation allow the recognition of an orbital lesion as inflammatory and the diagnosis of the underlying inflammatory disease. All in all, a proper treatment can be established, and at times, a biopsy can be avoided.

## Introduction

Orbital inflammation may be either idiopathic or in the context of a specific inflammatory disease. It may involve different orbital structures, accounting for the different clinical presentations. Recognizing the inflammatory etiology of a lesion, identifying which structures are involved, and determining the underlying disease is mandatory in order to establish an adequate treatment [1].

The diagnosis of orbital inflammation is made through combining the radiological findings, laboratory data, and characteristics of other organ involvement. When the diagnosis still remains unclear, tissue characterization and/or a therapeutical test is needed.

Despite its ability to identify orbital pathology as inflammatory and allowing for a specific diagnosis, imaging findings are often mistaken for infection and tumor [1–7].

This may be due to a number of reasons, the lack of detailed studies concerning the differential diagnosis on radiological imaging of orbital inflammatory diseases being one.

The purpose of this manuscript is to provide a comprehensive review of orbital inflammation, together with a systematic approach for the radiological evaluation of these patients, in order to improve the diagnostic accuracy of orbital inflammation.

CT and MRI protocols will be addressed first. Secondly, the specific radiological characteristics of inflammation affecting the various orbital structures will be illustrated, providing the necessary clues to differentiate orbital inflammation both from orbital infection and tumor. Thirdly, the imaging characteristics of specific inflammatory diseases will be presented, emphasizing the main features that will allow differentiation between distinct etiologies. Finally, a decision tree, combining mainly imaging features and clinical findings, will be provided, which will help in the differential diagnosis of orbital inflammatory diseases.

## Imaging protocols

MRI is the modality of choice for the evaluation of orbital inflammation because of its superior soft tissue contrast and spatial resolution, as well as its possibility to generate functional images such as diffusion-weighted imaging (DWI) and perfusion-weighted imaging (PWI).

Orbital lesions should be evaluated in multiple planes, preferably at least in axial and coronal planes. However, when a lesion is located in the eyelid, in the region of the posterior wall of the globe, or in close relationship with the optic nerve, additional sagittal oblique images should be obtained for an optimal evaluation.

In general, orbital evaluation with MRI is performed by using a head coil. The MRI protocol should include T1-weighted imaging (WI) sequences and T2-WI sequences with and without a fat suppression technique, T1-WI sequences with a fat suppression technique after contrast medium administration and DWI. T1-WI and T2-WI are the standard anatomical images to be obtained. They are important to determine which orbital structures are involved and to what extent. Inflammatory lesions are hypo- to isointense on T1-WI. On T2-WI sequences, the signal intensity of inflammatory lesions depends on the balance between edema and fibrosis, edema being hyperintense and fibrosis hypointense. The use of T2-WI sequences with fat suppression will make edema more conspicuous. Fat-suppression techniques after contrast sequences allow for the differentiation between an abnormal enhancing lesion and the normal bright signal of fat on T1-WI. Enhancement pattern can be important in differentiating inflammation from tumor and infection. Inflammatory lesions will tend to show a more homogeneous enhancement pattern while tumors and infections will be more heterogeneous due to the presence of non-enhancing components such as necrosis and pus. DWI can be performed using either echo planar imaging (EPI) or non-EPI-based sequences. An EPI-based sequence is the traditional choice for DWI. It has high temporal resolution but is sensitive to susceptibility artifacts and image distortion, especially present at air-tissue and bone-tissue interfaces, making it a challenging technique in the orbit. A non-EPI technique takes longer but does not show image distortions and susceptibility artifacts. DWI helps distinguish benign from malignant lesions. In the study from Sepahdari et al. with 189 cases, orbital masses were likely to be malignant (> 90% probability) when ADC < 0.93 × 10^–3^ mm^2^/s and likely to be benign (> 90% probability) when ADC > 1.35 × 10^–3^ mm^2^/s. Inflammatory lesions due to its higher free water content will have less diffusion restriction and therefore will show high ADC values. Meanwhile, malignant tumors having higher cellular content will restrict water diffusion and show low ADC values. In cases of bacterial infection, the presence of pus will be responsible for restricting diffusion and consequently high signal on DWI, matching the non-enhancing portion of the mass [8–10]. PWI can be performed in the orbit but few studies have been published. Most used a dynamic contrast-enhanced technique (DCE) in which serial T1-weighted images are acquired before, during, and after contrast administration. It provides data in the wash-in and wash-out contrast kinetics within a lesion. In DCE-MRI, the qualitative evaluation of the time intensity curve (TIC) pattern seems to be a complementary investigation in distinguishing benign from malignant lesions. In the study from Yuan et al., a persistent TIC pattern (type I curve) suggests a benign lesion, a wash-out TIC pattern (type III curve) mostly suggests malignancy, and a plateau TIC pattern (type II curve) occurs both in benign and malignant lesions [11].

In an emergency setting, computed tomography (CT) is often the first-line imaging modality because of its availability, high temporal resolution, and allowing oftentimes the diagnosis of a mass lesion. It may also identify a metallic foreign body that could become harmful during MRI examination. However, CT diagnostic performance compares negatively with MRI, namely in the differentiation between inflammation and tumor, as it lacks the information obtained through DWI and due to its worse soft tissue contrast and spatial resolution.

Ultrasonography is another alternative imaging method to diagnose inflammation or tumors of the globe in selected cases, but the technique is operator dependent and shows limited capacity in the evaluation of the retrobulbar structures [12, 13].

## Characteristics of orbital inflammation involving different orbital structures

Although infection may cause inflammation, in this paper, when referring to inflammatory disease, we are considering non-infectious inflammation.

Orbital inflammation may involve one or several orbital structures [1]. Table 1 summarizes the main imaging features in inflammation related to the different orbital structures.

**Table 1:** Imaging characteristics of inflammation involving different orbital structures

Orbital inflammation/orbital structure	Imaging characteristics
Scleritis (Fig. 1)	Scleral enhancement, scleral thickening, no DWI restriction, focal periscleral cellulitis
Uveitis (Fig. 2)	Uveal tract increased enhancement, uveal tract thickening, no DWI restriction, subretinal effusions, vitreous humor signal abnormalities
Dacryoadenitis (Fig. 3)	Lacrimal gland enlargement both involving the orbital and palpebral lobes, no DWI restriction, surrounding cellulitis
Optic perineuritis (Fig. 4)	Optic nerve sheath enhancement, no DWI restriction, surrounding cellulitis
Optic neuritis	Optic nerve enhancement and hyperintensity on T2 and FLAIR, DWI restriction possible
Myositis (Fig. 5)	Muscle enlargement, with increased enhancement, no DWI restriction, tubular/fusiform configuration, surrounding cellulitis
Cellulitis (Fig. 6)	Preseptal fat thickening, pre and postseptal fat infiltration and enhancement, no DWI restriction

**Fig. 1:**
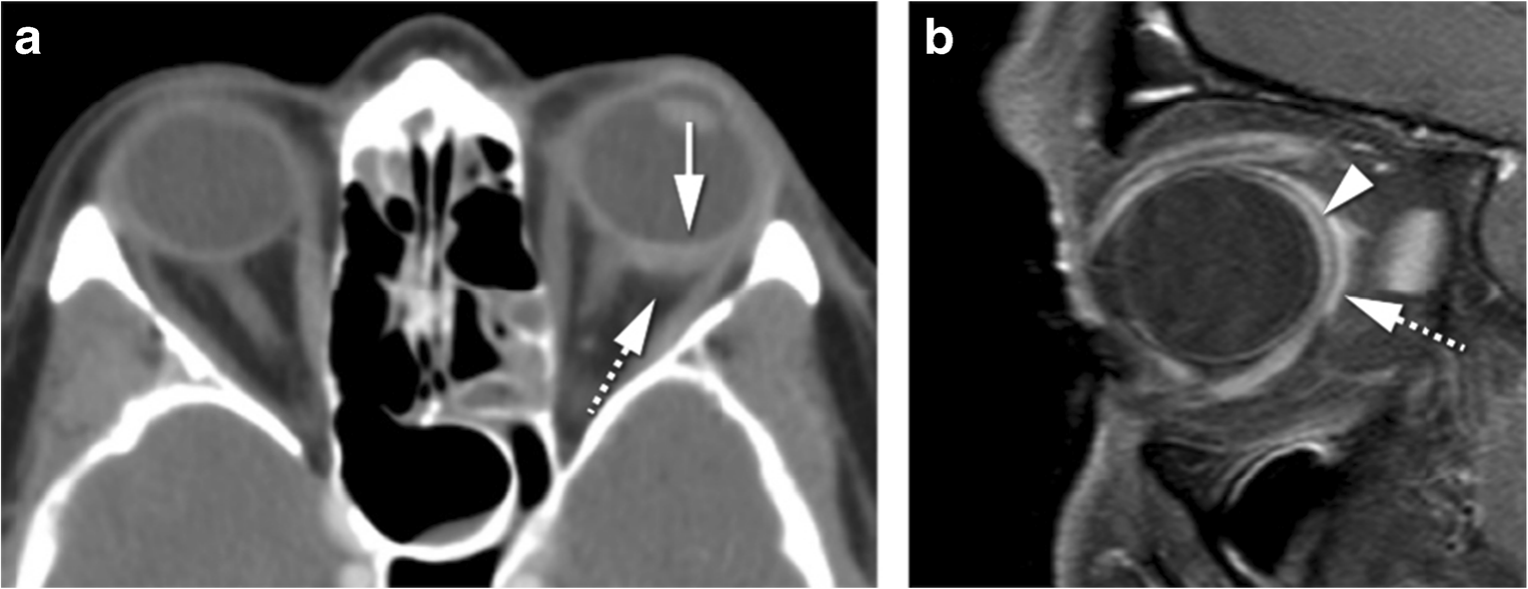
**a** 69-year-old female. Inflammatory scleritis on the left. Axial contrast enhanced CT (CECT): focal eccentric outward thickening and enhancement of the globe wall (arrow) and minimal blurring of the adjacent fat (dashed arrow), both consistent with scleritis. **b** 38-year-old male. Idiopathic inflammatory scleritis on the right. Enhanced sagittal T1-WI with fat signal suppression: scleral thickening and enhancement (dashed arrow) and slight suprachoroidal effusion (arrowhead), consistent with scleritis. Contrary to CT, MRI clearly depicts which ocular layer is enhancing

**Fig. 2:**
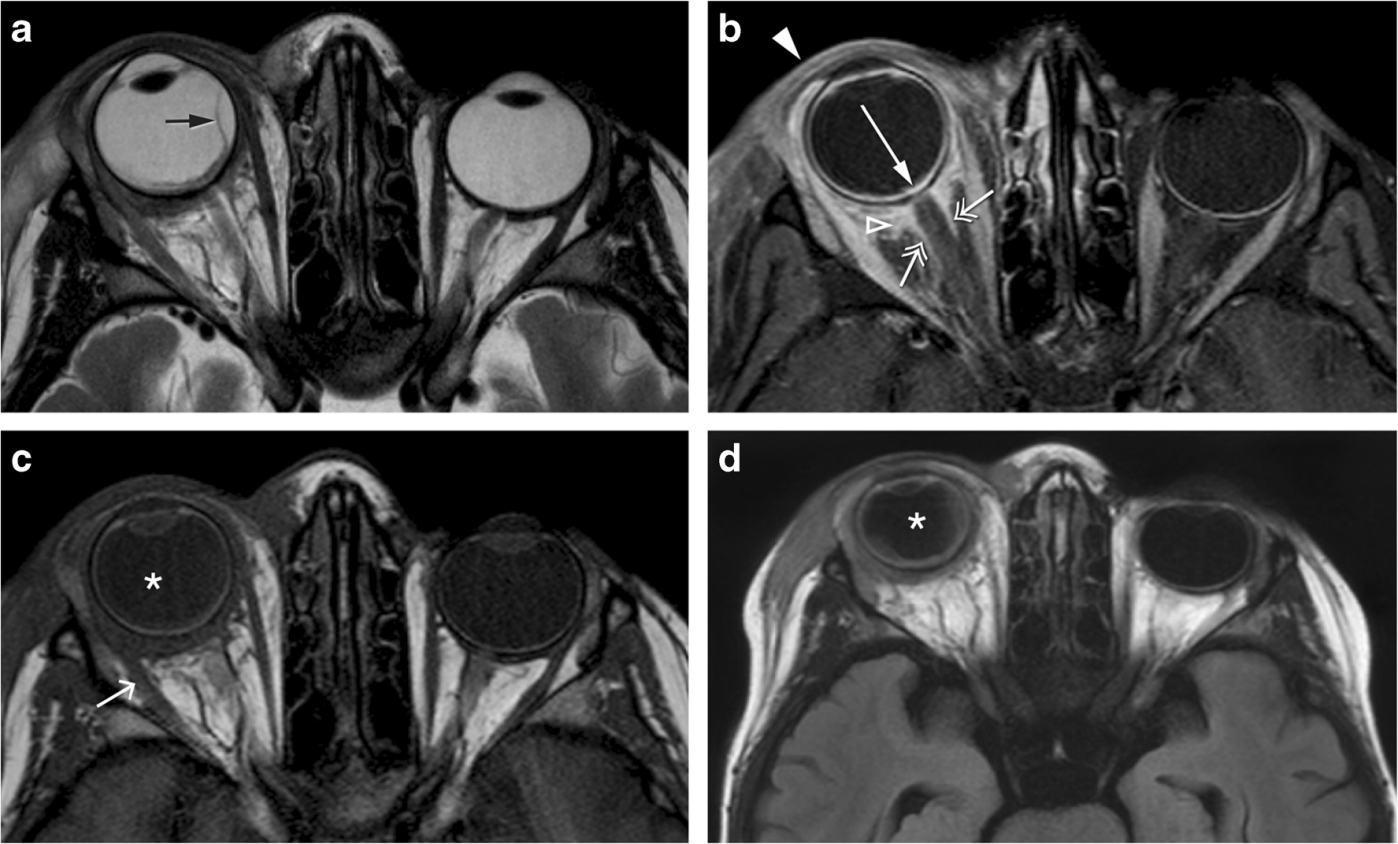
8-year-old male with graft versus host disease after stem cell transplant. Extensive orbital cellulitis, myositis involving the lateral rectus, and sclerouveitis on the right. Axial T2-WI (**a**), axial T1-WI (**c**), enhanced axial T1-WI with fat signal suppression (**b**), and axial FLAIR (**d**): increased thickness and enhancement of the whole uvea consistent with uveitis (long arrow), subretinal effusions (black arrow), and slightly increased signal intensity of the vitreous humor signal on T1 and FLAIR consistent with vitritis (asterisk). Extensive periscleral cellulitis due to scleritis (empty arrowhead), preseptal cellulitis (arrowhead), myositis of the lateral rectus (short arrow) and perioptic neuritis (double headed arrows)

Scleritis is an inflammation or infection of the sclera. The most common etiology is inflammatory, either idiopathic (43%) or in the context of a systemic disease (48%), most usually rheumatoid arthritis or granulomatosis with polyangiitis [12, 14]. Infectious scleritis is rare (7%) and is associated with predisposing factors such as surgery or trauma [11]. The diagnosis of scleritis is usually based on clinical assessment with intense and localized pain, with or without choroidal folds on fundoscopy and scleral thickening on ultrasonography. Image evaluation should be performed with MRI. MRI findings are quite distinct and include scleral enhancement, scleral thickening, and focal periscleral cellulitis, with no DWI restriction. Scleritis can be diffuse or less common nodular, the latter with a mass-like lesion in the wall of the globe (Fig. 1) [12]. Uveitis is an inflammation or infection of any part of the uveal tract, comprising the iris anteriorly, the ring-shaped ciliary body, and the choroid posteriorly. When inflammatory, uveitis is either idiopathic or in the context of a systemic disease, such as HLA-B27-associated seronegative spondylarthropathies, rheumatoid arthritis, and sarcoidosis [15]. The diagnosis of uveitis is predominantly based on clinical symptoms, such as pain, photophobia, decreased vision, and, on slit-lamp biomicroscopy and fundoscopy, the presence of cells and flare in the vitreous and anterior chamber respectively. If image evaluation is considered, it should be through MRI. MRI signs of uveitis include increased enhancement and/or thickening of the uveal tract, subretinal effusions, and vitreous humor signal abnormalities, with no DWI restriction (Fig. 2) [15]. On imaging, inflammatory scleritis and infectious scleritis are alike, as are inflammatory and infectious uveitis. Inflammatory scleritis looks similar whatever the underlying inflammatory disease is, the same holding true when inflammatory uveitis is considered. Nodular scleritis and nodular uveitis need to be differentiated from a tumor [16, 17]. On both cases, the presence of pain and lack of diffusion restriction on MRI should raise the suspicion of an inflammatory or infectious condition. Moreover, the presence of periscleral cellulitis and a located scleral mass both favor the diagnosis of nodular scleritis over tumor [12].

**Fig. 3:**
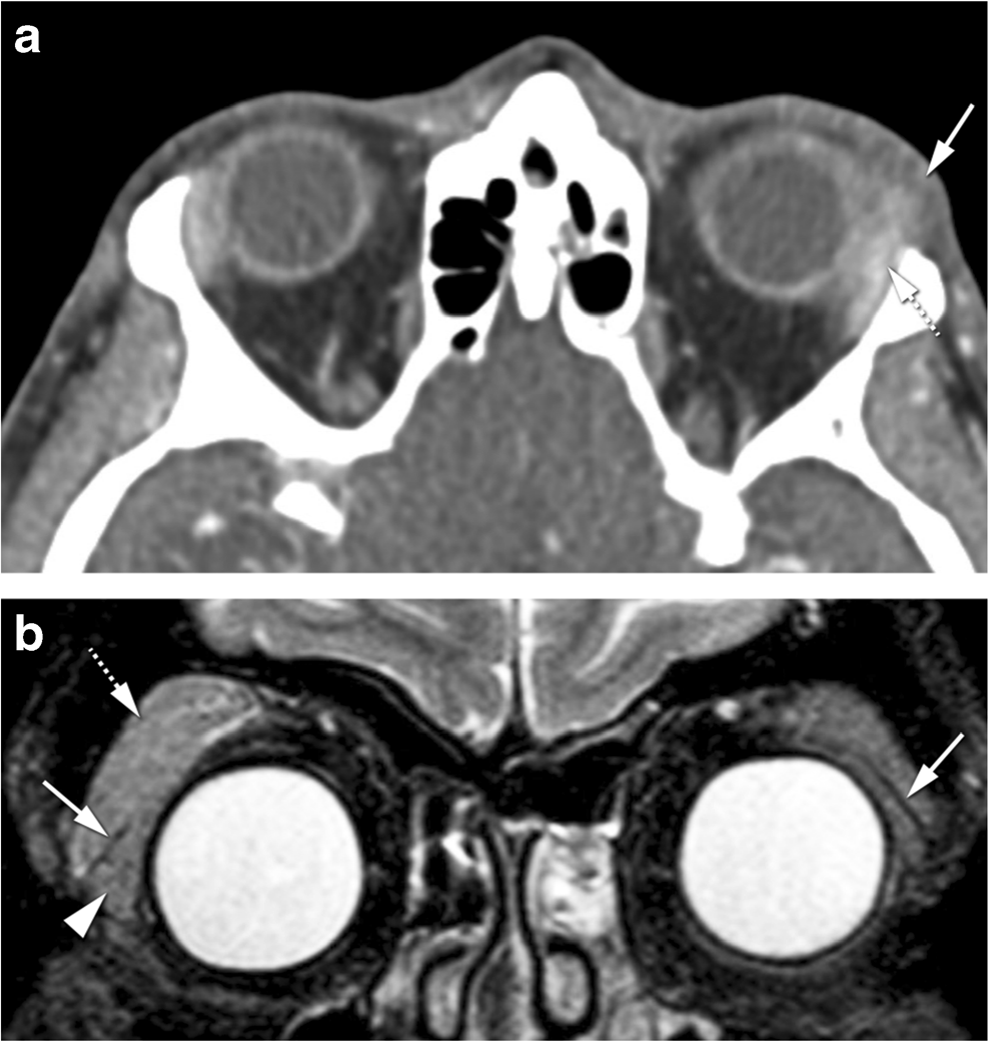
**a** 68-year-old male with Stills’ disease. Inflammatory dacryoadenitis on the left. Axial CECT: enlarged lacrimal gland (dashed arrow) with slight blurred margin and preseptal cellulitis (arrow) consistent with dacryoadenitis. The coexistence of preseptal cellulitis makes the diagnosis of tumor not probable. **b** 54-year-old female with Sjögren’s disease. Bilateral inflammatory dacryoadenitis. Coronal T2-WI with fat signal suppression: bilateral enlargement of the lacrimal gland, involving both the orbital (dashed arrow) and palpebral (arrowhead) lobes, a feature typical of dacryoadenitis but that can also occur in lymphomas. The levator palpebrae tendon separating the orbital and palpebral lobes (arrows)

Dacryoadenitis is an inflammation or infection of the lacrimal gland. It can be acute such as due to infection or idiopathic orbital inflammation or chronic related to sarcoidosis, Sjögren’s syndrome, granulomatosis with polyangiitis, Graves’ disease, and IgG4-related disease. Clinically, it is characterized by swelling of the lateral third of the upper eyelid, as well as redness and, especially in case of acute presentation, pain. On CT and MRI, a diffuse enlargement of the gland is observed, maintaining its normal almond shape and involving both the orbital and palpebral gland lobes [2]. Surrounding cellulitis is possible, with blurring of the glandular margin and possibly involving the adjacent muscles [1, 2, 18]. There can be a tapered posterior margin of the gland until the apex (Fig. 3a) [2]. On imaging, inflammatory and infectious dacryoadenitis look similar. Furthermore, inflammatory dacryoadenitis looks similar whatever the underlying cause is. Dacryoadenitis must be differentiated from a tumor. A benign tumor will be confined to the orbital lobe, with no surrounding cellulitis and no invasion of adjacent structures [2]. A malignant tumor has no surrounding cellulitis and has diffusion restriction. In cases of infectious dacryoadenitis with abscess formation, restricted diffusion is also expected. Lymphomas can, just as dacryoadenitis, involve both the orbital and palpebral lobes and have a tapered posterior margin until the apex, but unlike dacryoadenitis, lymphomas have diffusion restriction. When dacryoadenitis is bilateral, the differential diagnosis includes idiopathic orbital inflammation, Graves’ disease, sarcoidosis, Sjögren’s syndrome, IgG4-related disease, or granulomatosis with polyangiitis (Fig. 3b) [19].

**Fig. 4:**
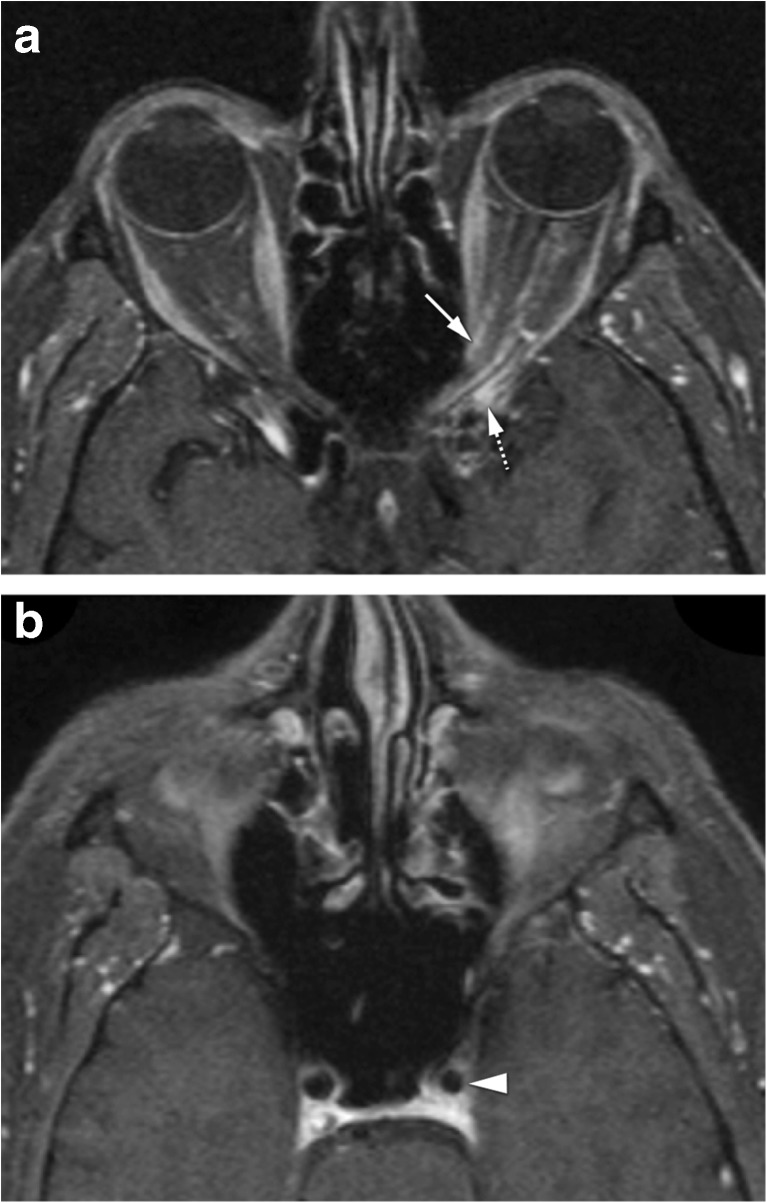
40-year-old male with Tolosa-Hunt disease. **a** Optic perineuritis on the left at the orbital apex. Enhanced axial T1-WI with fat signal suppression: tram-track sign (arrow) and slight streaky enhancement of the surrounding fat consistent with perioptic neuritis. Slight orbital apical enhancement (dashed arrow) keeping with Tolosa Hunt disease. **b** Enhanced axial T1-WI with fat signal suppression at the level of the carotid siphons: smaller internal carotid artery on the left (arrowhead), due to pericarotid inflammatory tissue, a known finding in Tolosa-Hunt disease

Optic perineuritis (OPN) and optic neuritis (ON) have been considered different entities, the first affecting the optic nerve sheath and the second the optic nerve itself [2, 20]. It is important to distinguish them because they tend to have different etiologies. OPN is either inflammatory, infectious, or idiopathic, while ON is demyelinating, idiopathic, or in the context of the neuromyelitis optica spectrum disorder [20, 21]. Clinically OPN is difficult to differentiate from ON, both presenting with vision loss, pain with eye movement, and either a normal or swollen optic disk. OPN has a broad age distribution with most patients being above 50 years old. ON tend to affect younger adults, especially females [20]. Image evaluation should be performed with MRI. In OPN, there is enhancement of the optic nerve sheath, with the characteristic tram-track sign on axial and sagittal images and the donut sign on coronal sequences. Surrounding cellulitis is a possible other feature in OPN (Fig. 4) [2, 20]. In ON, the enhancement and hyperintensity on T2 and FLAIR sequences are located in the nerve itself [20]. This differentiation may also have important therapeutic and prognostic implications [21]. With the potential serious side effects associated with high-dose systemic steroid therapy, some clinicians may choose not to prescribe steroids for typical multiple sclerosis-related ON, particularly as steroid therapy has been shown not to positively affect long-term visual acuity [21]. On the other hand, early initiation of steroid treatment for OPN is essential to prevent irreversible visual loss and recurrence. OPN typically does not naturally resolve [21]. ON must be distinguished from optic ischemic neuropathy as well. Differentiation is not straightforward. Enhancement is more commonly seen with ON while lower ADC is more typical of ischemic neuropathy [22, 23]. OPN must be distinguished from meningioma, the latter being usually thicker, may show calcifications and there may be hyperostosis of the optic canal. Meningiomas will not typically respond to steroids [2].

**Fig. 5:**
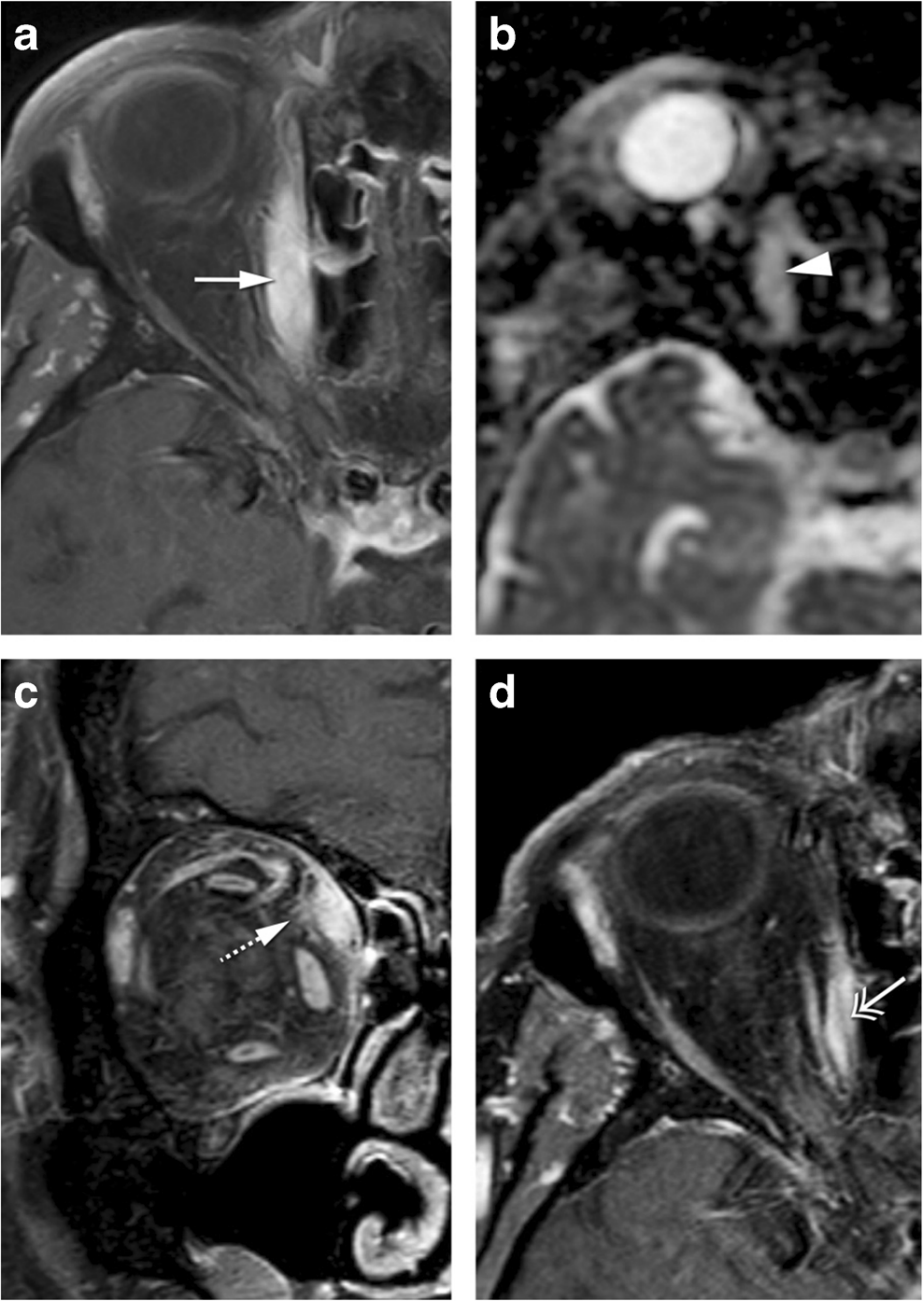
68-year-old male. Inflammatory myositis of the right superior oblique muscle. Enhanced axial T1-WI with fat signal suppression (**a**, **d**), axial ADC (**b**), and enhanced coronal T1-WI with fat signal suppression (**c**): enlarged and with marked contrast enhancement right superior oblique muscle (arrow), with no DWI restriction (arrowhead), and slight surrounding cellulitis (dashed arrow), favoring inflammation. Clinically not suspicious for infection. Improvement after corticosteroids (**d**) avoiding biopsy (double-headed arrow)

Myositis is an inflammation or infection of the muscle. One or more muscles can be simultaneously involved. Clinically, it is characterized by prominent pain especially with eye movement. On CT and MRI, the involved muscle/s is enlarged, there is too much enhancement, and in case MRI is performed, no diffusion restriction is expected. Surrounding cellulitis is possible and the signal on T2-WI is variable depending on the etiology (Fig. 5). On imaging, an inflammatory myositis looks similar to an infectious myositis. In case of an inflammatory myositis, some clues will point the underlying inflammatory etiology. Idiopathic orbital inflammation involves especially the medial, followed by the superior and lateral recti; the muscle has a tubular configuration and there is frequently surrounding cellulitis [2, 18]. Graves’ disease is typically bilateral, the superior oblique and lateral recti are relatively spared and the muscles have a fusiform configuration [2]. On IgG4-related disease, muscle involvement is typically bilateral, the lateral rectus is the most commonly affected, muscles have a fusiform aspect and there can be surrounding cellulitis [24]. A myositis also needs to be differentiated from a tumor such as lymphoma or metastasis. The presence of pain, surrounding cellulitis, and the absence of diffusion restriction are suggestive of inflammation or infection [2].

**Fig. 6:**
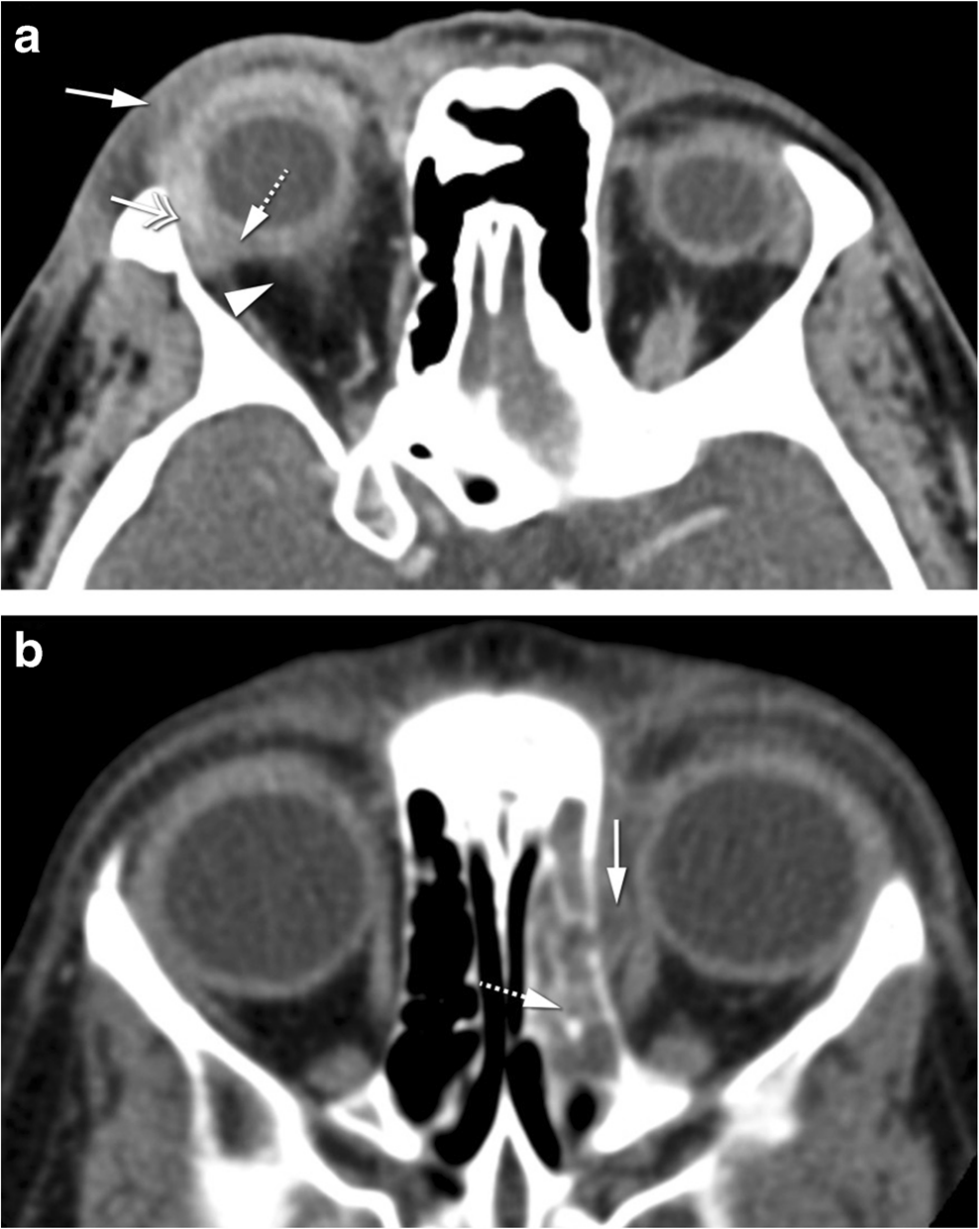
**a** 46-year-old female with idiopathic orbital inflammation on the right. Axial CECT: pre (arrow) and postseptal (arrowhead) cellulitis, scleritis (dashed arrow), and dacryoadenitis (double-headed arrow). Scleritis and no sinusitis favored inflammation over infection. **b** 5-year-old male. Axial CECT: pre- and postseptal cellulitis on the left. Ethmoiditis (dashed arrow) and subperiosteal abscess (arrow), both favoring an infectious etiology

Orbital cellulitis refers to orbital fat inflammation or infection. It can be pre- or postseptal [2]. Clinically, preseptal cellulitis presents with swollen, reddish, and painful eyelids, while in postseptal cellulitis proptosis, restriction of eye movements and disturbed pupillary reflexes may exist. Signs of cellulitis are easy to appreciate on CT and MRI and include thickening of the fat, best appreciated on the preseptal space, fat infiltration, and contrast enhancement. On MRI, the signal on T2-WI is variable depending on the etiology. Inflammatory cellulitis is frequently confused with infectious cellulitis. On imaging, the presence of sinusitis or abscess suggests infection, while the presence of scleritis points to inflammation (Fig. 6) [2, 12].

**Fig. 7:**
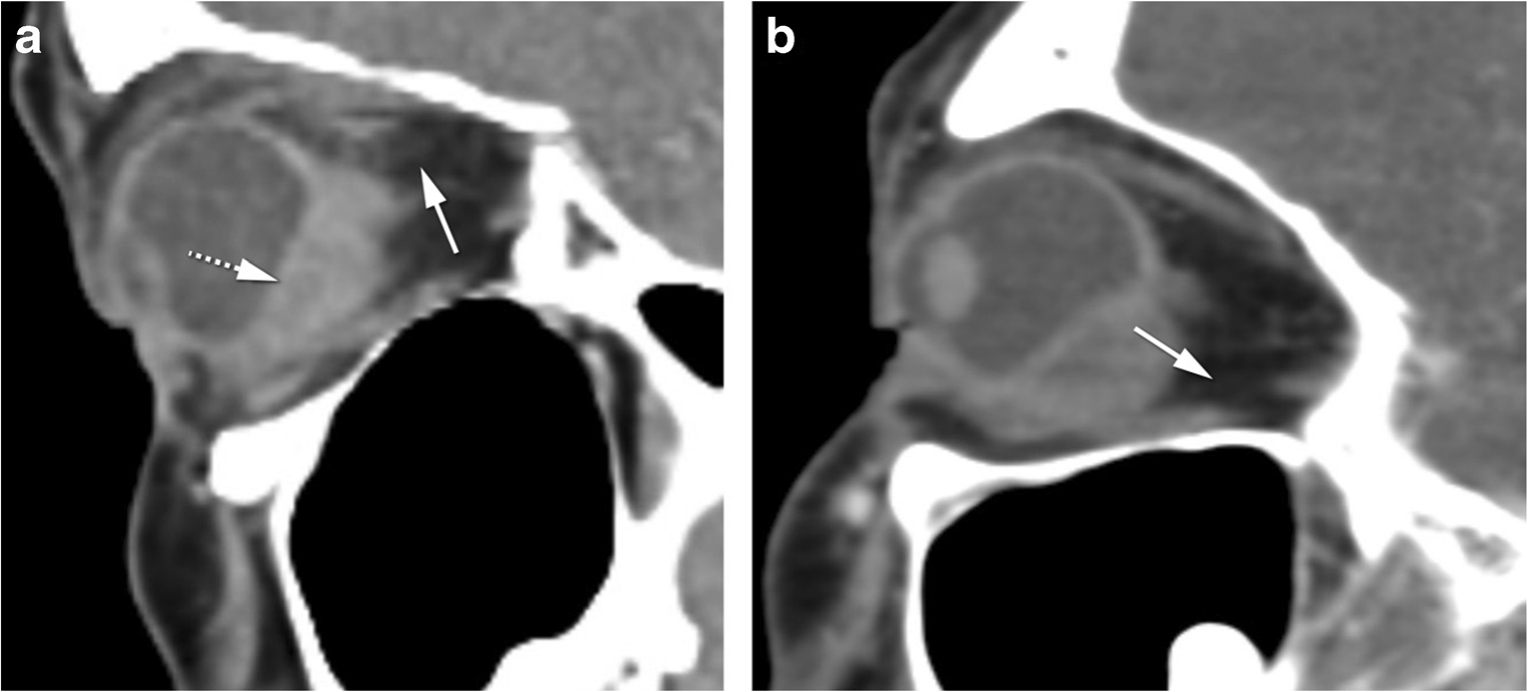
**a** 41-year-old female. Idiopathic inflammatory nodular scleritis on the left. Sagittal CECT: posterior wall globe focal mass. Deviation of the choroid-retinal layer internally (dashed arrow) suggesting a scleral or periocular origin. Painful periscleral cellulitis (arrow) favoring the diagnosis of scleritis. **b** 57-year-old female. Periocular breast cancer metastasis. Sagittal CECT: posterior wall globe focal mass, similar to (**a**), but note the normal aspect of the fat (arrow). Clinically there was no pain. Both favoring the diagnosis of a tumor

Inflammation will sometimes present as a focal solid-enhancing mass, which can be located anywhere in the orbit [2]. There is no diffusion restriction and surrounding cellulitis may exist. The clinical symptoms and signs are dependent of the anatomical location of the mass, but pain is frequently present. The main differential is tumor [2, 12]. The presence of pain, surrounding cellulitis, and absence of diffusion restriction will point to inflammation. As has been said, if the mass is in the globe wall originating from the sclera, the diagnosis of inflammation will be favored (Fig. 7) [12].

## Characteristics of orbital inflammation due to different inflammatory diseases

Orbital inflammation can have a wide range of underlying etiologies. It can be idiopathic, or in the context of Graves’ disease, sarcoidosis, granulomatosis with polyangiitis, IgG4-related disease, or sclerosing orbital inflammation [25]. More rarely, it is associated with other systemic diseases such as Erdheim-Chester [1]. Over the past decade, therapeutic options in orbital inflammatory disease have evolved markedly, from prednisone and cyclophosphamide, still widely used, to targeted immunotherapy. Treatment is becoming more and more etiology specific, and therefore, recognizing the underlying disease in orbital inflammation has strong clinical implications. In Table 2, the main features of orbital inflammation due to different inflammatory diseases are summarized.

**Table 2:** Main imaging characteristics of the most common orbital inflammatory diseases

Orbital inflammatory disease	Main imaging characteristics
IOI (Fig. 8a–d)	Pain, can involve all orbital structures, myositis with tubular configuration, cellulitis
Sarcoidosis (Fig. 8e)	Similar to IOI but: pain unusual, uveitis most common manifestation, predilection for antero-inferior quadrant
Graves’ D (Fig. 9a, b)	Bilateral, myositis with predilection for inferior and medial quadrant and with fusiform configuration, increased orbital fat, no cellulitis
IgG4 RD (Fig. 9c–e)	Bilateral, chronic course, predilection for lateral and superior quadrant with myositis and dacryoadenitis, myositis with fusiform configuration, cellulitis, infraorbital nerve involvement
Granulomatosis with polyangiitis (Fig. 10)	Predilection for extraconal and conal compartments, chronic sinonasal involvement with bone destruction
ISOI (Fig. 11)	Chronic course, predilection for lateral and superior quadrant with myositis and dacryoadenitis, enophthalmus possible

*IOI* idiopathic orbital inflammation *Graves’ D* Graves’ disease, *IgG4 RD* immunoglobulin G4-related disease, *ISOI* idiopathic sclerosing orbital inflammation

**Fig. 8:**
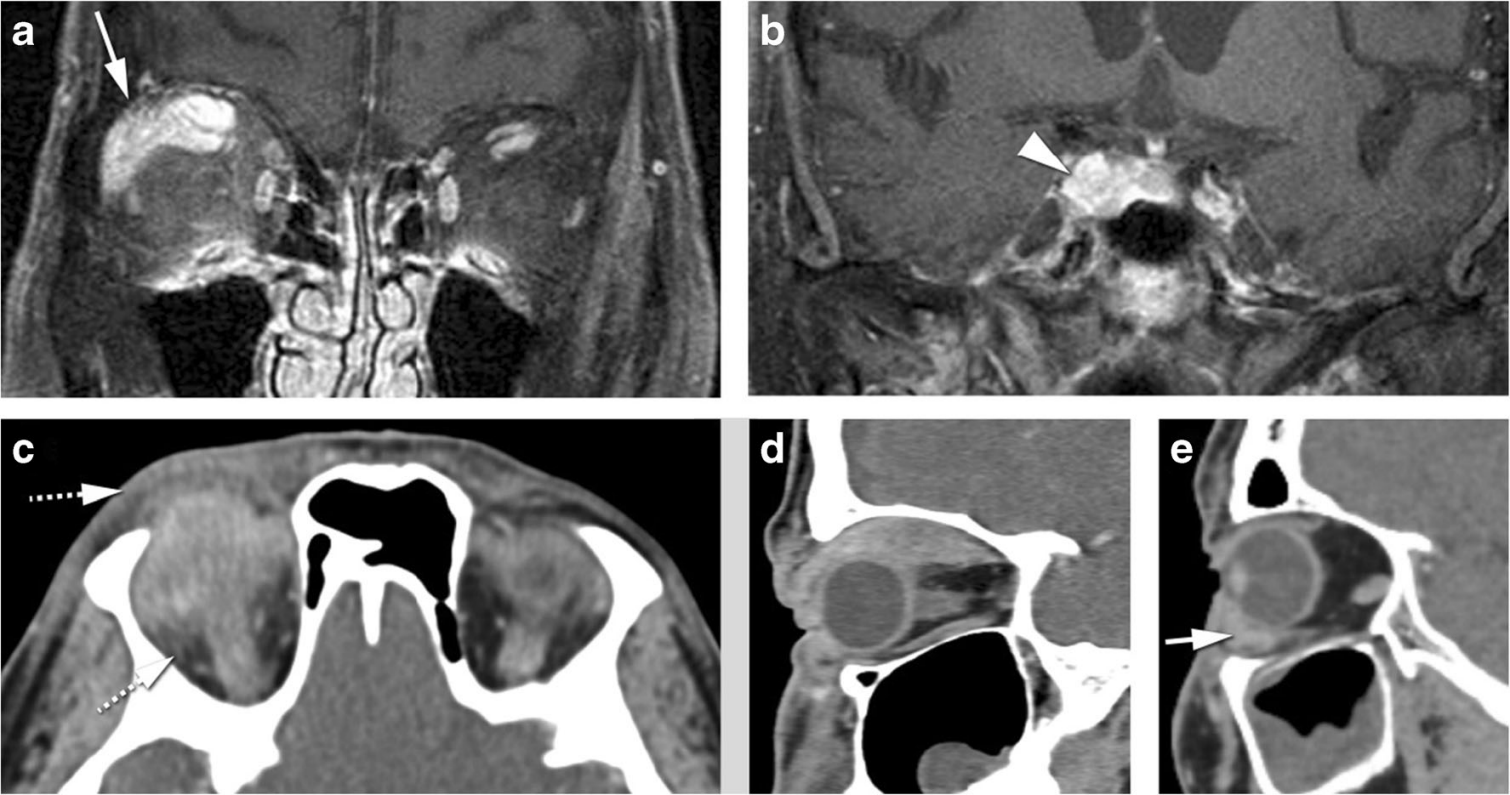
**a, b** 89-year-old male with idiopathic orbital inflammation. Enhanced coronals T1-WI with fat signal suppression (**a**, **b**): unilateral multifocal disease on the right involving the complex levator palpebrae-superior rectus and lacrimal gland (arrow) and the cavernous sinus (arrowhead). The bright signal around the inferior recti is due to inhomogeneous fat saturation. **c, d** 46-year-old man with idiopathic orbital inflammation. Axial (**c**) and sagittal (**d**) CECT: myositis of the right superior muscle complex with surrounding cellulitis (dashed arrows). Muscle involvement encompasses tendon and muscle belly giving the muscle a tubular configuration (**d**). **e** 43-year-old male with sarcoidosis. Sagittal CECT: typical involvement of the antero-inferior quadrant of the left orbit with mass in the inferior eyelid (arrow)

Idiopathic orbital inflammation (IOI), often called pseudotumor, is a diagnosis of exclusion, and therefore, infection, malignancy, and a systemic inflammatory process must be ruled out [2]. Although the cause of IOI is unknown, an immune-mediated pathophysiological mechanism is likely [18]. It is the third most common orbital disease after Graves’ orbitopathy and lymphoproliferative disorders [2, 18]. Generally, acute IOI presents with pain and is unilateral for most of the times (75%) [2, 18]. Imaging shows an infiltrative mass, less often a focal mass, most of the time hypointense on T2-WI, with contrast enhancement [18] and lacks diffusion restriction [8]. IOI can involve any orbital compartment, commonly involving multiple sites at the same time [18]. The preseptal space is however less involved compared to lymphoma [10]. In its muscular form, IOI affects especially the medial followed by the superior and lateral recti [18, 24], involving both the belly and the tendon, giving the muscle a tubular appearance. Most of the time, the surrounding fat is involved (Fig. 8a–d) [2, 18]. Fibrosis is frequently present at pathological examination [18]. Treatment for IOI consists of intravenous steroids [2]. Radiotherapy and chemotherapeutic agents such as methotrexate are alternative treatments [18]. Tolosa-Hunt syndrome is a rare subtype of pseudotumor with involvement confined to the orbital apex and/or cavernous sinus resulting in acute orbital pain and paralysis of cranial nerves III, IV, V (superior division), and VI [2]. The orbital pain should resolve within 72 h when treated adequately with steroids, ophthalmoparesis usually requiring a little longer depending on inflammation degree and steroids regimen.

Sarcoidosis is a systemic inflammatory disease of unknown etiology, characterized by the presence of granulomas in the affected organs [26]. Lungs and skin are most commonly affected. Sinonasal involvement is rare [27]. Orbital involvement is seen in 25–60% of patients with systemic sarcoidosis [28]. Sarcoidosis can involve any orbital compartment [28], similar to IOI. However, in sarcoidosis, uveitis is the most common manifestation, the antero-inferior orbital quadrant is involved to a greater degree, the cavernous sinus can be affected as well and isolated myositis is rare (Fig. 8e) [26]. The involved orbital structures are hypointense on T2, enhance and no DWI restriction is expected. Clinical presentation is subacute evolving from months to years. Pain is not a typical feature. Orbital involvement is unilateral in 75% of cases. Isolated orbital granulomatous involvement, in the absence of systemic disease, should not be called orbital sarcoidosis, as it may represent an idiopathic granulomatous orbital inflammation, probably a different entity, affecting especially men in the fourth decade and in 50% of cases affecting the lacrimal gland [26, 29]. Serum angiotensin-converting enzyme (ACE) is increased in 60 to 90% of patients with active disease and reflects its severity [1, 30]. Oral steroids are the mainstay of treatment. Cytotoxic agents (v.g. methotrexate) are used as second line. Surgical excision may be considered for localized orbital disease, namely the eyelid [26].

**Fig. 9:**
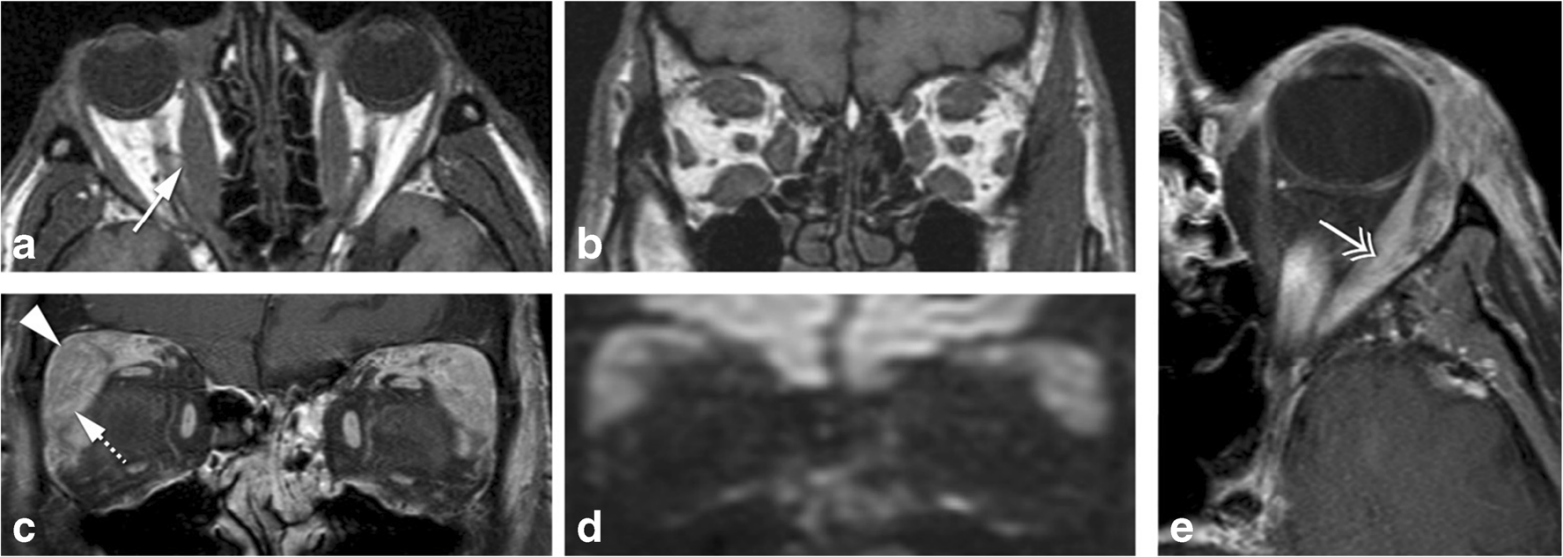
**a**, **b** 40-year-old female with thyroid-associated orbitopathy. Axial (**a**) and coronal T1-WI (**b**): bilateral and symmetric enlargement of the inferior, medial and complex levator palpebrae-superior rectus muscles. Notice fusiform configuration of the muscles because sparing of the tendon (arrow) and no surrounding infiltration of the fat. **c**–**e** 35-year-old male with IgG4-related disease. Coronals enhanced T1-WI with fat signal suppression (**c**) and DWI (**d**) and axial enhanced T1-WI with fat signal suppression (**e**): bilateral involvement of the superolateral quadrants of the orbits, including bilateral enlargement of the lacrimal glands with involvement of some of the adjacent muscles. Involvement of both the orbital (arrowhead) and palpebral (dashed arrow) lobes of the lacrimal gland. Fusiform configuration (double-headed arrow) of the muscle. No diffusion restriction favoring an inflammatory process

Thyroid-associated orbitopathy (TAO) is an autoimmune condition of the orbit, more often associated with Graves’ hyperthyroidism, but it may exist in patients with euthyroid or hypothyroid chronic autoimmune thyroiditis [31]. The most important pathogenic factors are the thyroid-stimulating hormone (TSH) receptor auto-antibodies, sharing as targets TSH receptors localized on orbital fibroblasts and adipocytes [31]. On TAO, the extraocular muscles are the most common orbital structure involved [4]. Muscle involvement is bilateral on 90% and symmetrical on 70% of cases. The inferior rectus muscle is usually the first to be involved, followed by the medial, superior, and the lateral recti and the oblique muscles. The tendon tends to be spared giving the muscle a fusiform configuration [4]. No surrounding cellulitis is seen. Increased orbital fat and dacryoadenitis may coexist. The enlarged muscles and increased fat will induce proptosis. There can be crowding of the apex with compression of the optic nerve [4]. Indirect but objective signs of crowding of the apex and optic nerve compression are the presence of an enlarged superior ophthalmic vein and intracranial fat prolapse [4]. Proptosis may induce stretching of the optic nerve (Fig. 9a, b). Serologic testing includes measuring serum TSH, T3, T4, TSH-r antibody, thyrotropin-binding inhibitory immunoglobulin, and thyroid-stimulating immunoglobulin [32]. Natural history of the disease depicts an active phase followed by an inactive phase. MRI is a valuable tool to distinguish both stages, by demonstrating edema within the extraocular muscles in the active stage, translated as hypersignal on the STIR sequence [4, 31, 33]. Immunomodulatory therapies are the treatment of choice but are only effective in the active phase of the disease and therefore should not be considered in patients with inactive TAO [4].

Immunoglobulin G4-related disease (IgG4 RD) is a recently described systemic inflammatory process of unknown etiology [24]. Any organ can be involved but there is a predilection for the orbits, salivary glands, lymph nodes, pancreas, and hepatobiliary system. Mickulicz disease, previously thought to be a subtype of Sjögren’s syndrome, is now considered part of the IgG4 RD [34, 35]. IgG4 RD of the orbit has an indolent chronic course with symptoms evolving on average for 45 months at time of diagnosis [26]. Pain is not a characteristic finding [24, 36]. On IgG4 RD of the orbit, the extraocular muscles are the most common orbital structure involved (89%). Myositis is mostly bilateral (88%). The lateral rectus is the most affected muscle (76%) and typically enlarged to the greatest degree. The tendon is spared in 96% of cases, giving the muscle a fusiform configuration [24]. The lacrimal gland is the second most common orbital structure involved (70%) and its involvement is more common bilateral (58%) (Fig. 9 c–e) [24, 34]. Cellulitis is present in 44%, either pre- or postseptal or uni- or bilateral [24]. Perineural involvement has been reported, mostly affecting branches of the trigeminal nerve, the infraorbital nerve being involved in 30% and mostly unilateral [24, 35, 37–39]. There is expansion of the foramina [39]. In 89% of patients with IgG4 RD, there is sinusal disease as well [24]. At imaging, orbital IgG4 RD lesions are diffuse or tumefactive, homogeneous, hypointense on T2-WI, enhancing, with no DWI restriction [37, 40]. Bone remodeling is possible [37]. Increased IgG4 levels in serum will help in making the diagnosis [40], but serum IgG4 can be normal in up to 40% of patients with biopsy-proven disease [32, 38]. The definitive diagnosis is histopathologic typically with abundant IgG4-positive plasma cells and fibrosis [38, 41]. Lymphoma can be a complication of IgG4-related disease [35, 36, 38]. Although the most effective therapy of IgG4-RD has yet to be defined, rituximab is a promising alternative to glucocorticoids [3, 42, 43].

**Fig. 10:**
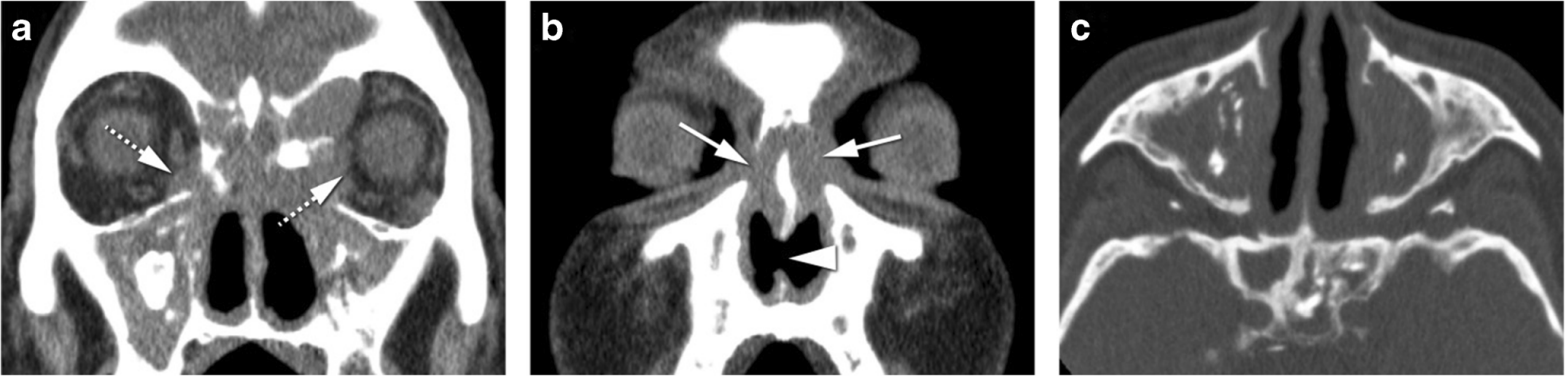
67-year-old male with granulomatosis with polyangiitis involving the orbit, nose, and sinuses. Coronals (**a**, **b**) and axial (**c**) CT: chronic pansinusitis with sclerosis of the bony walls and intrasinusal calcifications coexisting with nasal septum (arrowhead) and lamina papiracea (arrows) erosions. Bilateral orbital involvement in the extraconal compartment (dashed arrows)

Granulomatosis with polyangiitis, previously known as Wegener granulomatosis, is presumed to be an autoimmune disease but its exact nature remains unknown [44]. The organs most commonly involved are the lungs (95%), the paranasal sinuses (90%), and the kidneys (85%) [6]. Orbital involvement usually presents several years after the onset of the disease [45] and is mostly unilateral (86%) [6, 45]. Pain is occasionally present [1, 44]. There are two types of orbital involvement, granulomatous disease, causing an inflammatory mass, and small-vessel vasculitis, causing among others scleritis, uveitis, and optic neuritis [7]. In 70% of the cases, the granulomatous orbital form represents an extension from sinusal disease [7]. Extraconal involvement is present in 88% of the cases, frequently with conal extension [7]. Intraconal involvement without extraconal involvement is rare being present only in 6% of the cases [7]. Lesions enhance, are hypointense on T2-WI [7] and no DWI restriction is expected. Nasosinusal involvement is characterized by mucosal thickening, bone destruction, and nasal septal perforation (Fig. 10). The differential diagnosis based on image would include invasive fungal sinusitis, which has a different clinical context, the latter occurring in the setting of an immunosuppressed patient. The definitive diagnosis is based on tissue biopsy (granulomas, necrosis, and vasculitis) [7, 44]. A positive antineutrophil cytoplasmic antibodies (ANCA) is a marker of disease activity. It is present in more than 90% of patients with active systemic disease but only in 32% of patients with a limited form of the disease [1, 7, 30]. Therapy is based on immunosuppressive agents such as cyclophosphamide and corticosteroids [7].

**Fig. 11:**
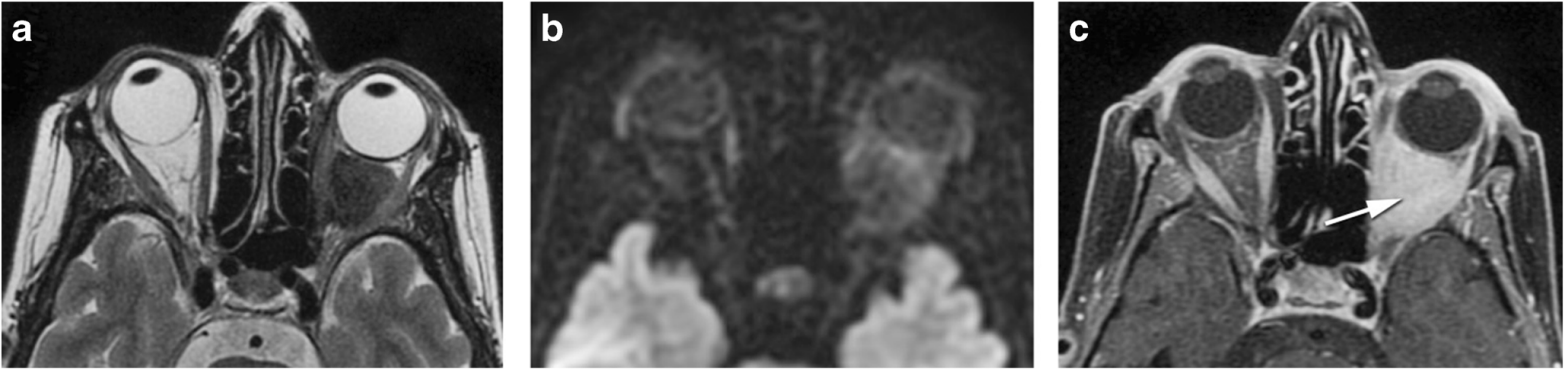
48-year-old female with idiopathic sclerosing orbital inflammation. Axial T2-WI (**a**), axial DWI (**b**) and enhanced axial T1-WI with fat signal suppression (**c**): large intraconal mass on the left (arrow), with no restriction diffusion, hypointense on T2, with marked enhancement after contrast. Notice enophthalmus, very unusual for a retrobulbar mass, together with absence of restriction diffusion, making it suspicious for sclerosing orbital inflammation

Idiopathic sclerosing orbital inflammation (ISOI) is a rare disease [45, 46]. Previously thought to be the endstage of IOI, it is now considered a distinct pathologic entity with marked fibrosis present early in the disease [45–47]. ISOI is a chronic, indolent process with symptoms evolving on average for 18 to 24 months at time of diagnosis. Pain may be present. On imaging, ISOI manifests as an ill-defined mass, slightly enhancing, hypointense on T2-WI, and with no DWI restriction. There is a predilection for the lateral and superior quadrants and therefore the lacrimal gland and the superior and lateral recti muscles are prone to be involved [45, 47]. Enophthalmus can exist due to the fibrotic process (Fig. 11) and extraorbital involvement is a possibility, with disease extending to the pterygopalatine fossa, to the masticator and buccal space, and to the intracranial compartment [45]. The diagnosis of ISOI depends largely on biopsy, showing marked fibrosis together with a mixed chronic inflammatory cell infiltrate [45]. First-line treatment, although still controversial, encompasses corticosteroids and azathioprine, with poor results as fibrosis increases and inflammation subsides [45]. Some patients may show an elevated seric and tissue IgG4 [46]. It is difficult to know whether IgG4-related orbital disease is a distinct entity or an additional defining characteristic of idiopathic sclerosing orbital inflammation [46]. Testing IgG4 is important as rituximab seems effective in IgG4-related disease. Differential diagnosis includes sclerosing lymphoma and sclerosing breast carcinoma metastasis [48], but these entities, unlike ISOI, will show restricted diffusion on MRI.

**Fig. 12:**
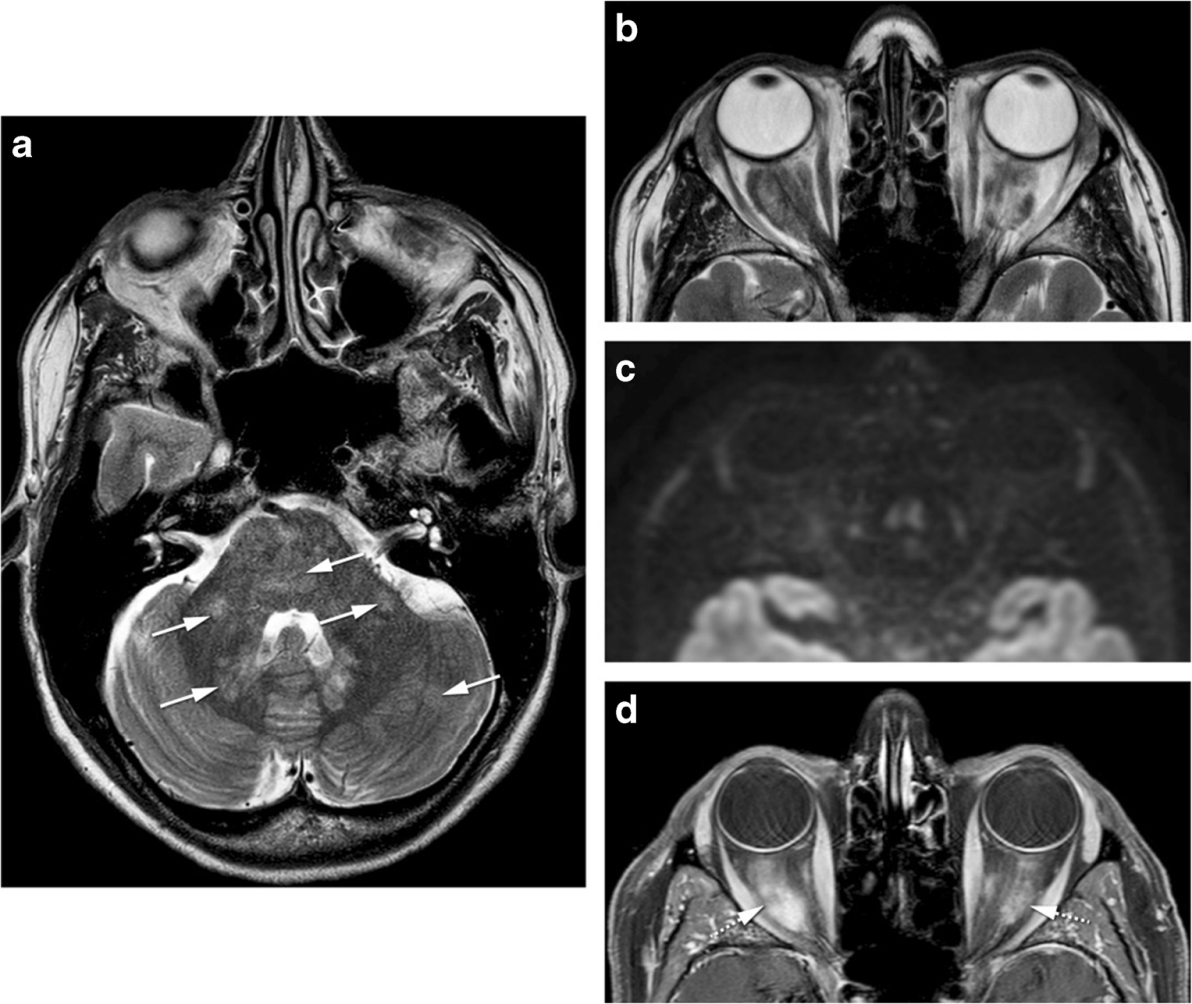
47-year-old female with Erdheim-Chester disease. Axial T2-WI (**a**, **b**), axial DWI (**c**) and enhanced axial T1-WI with fat signal suppression (**d**): bilateral intraconal masses surrounding the optic nerves (dashed arrows), slightly heterogeneous on T2, no diffusion restriction, enhancing after contrast. Bilateral involvement of cerebellum, middle cerebellar peduncles, and pons (arrows)

Erdheim-Chester disease is a rare non-Langerhans cell histiocytosis of unknown origin, with multiorgan infiltration by lipid-laden histiocytes, belonging to the group of xantogranulomatous diseases [49, 50]. The skeleton is the most commonly involved organ (96%), along with the brain, heart, lung, liver, kidney, skin, and retroperitoneal space [1]. Orbital involvement is also common. On imaging, Erdheim-Chester disease presents with infiltrative intraconal masses, hypointense on T2-WI, enhancing after gadolinium, and DWI restriction is not expected (Fig. 12) [49]. Xanthomatous lesions of the eyelids are also present. Histologic evaluation reveals foamy cell infiltration, Touton giant cells and fibrosis. Immunologic staining confirms the diagnosis as these cells are positive for CD68, a histiocytic marker [1].

## Decision tree in orbital inflammation

Orbital inflammation presents on radiological imaging as a solid-enhancing lesion, mostly as an ill-defined or infiltrative lesion. The differential diagnosis of an orbital solid-enhancing lesion is however vast including not only inflammation but also infection, benign and malignant tumors, and vascular malformations (e.g., cavernous hemangioma). In the presence of an orbital enhancing solid mass one should first recognize its inflammatory nature and second try to determine the underlying inflammatory disease.

**Fig. 13:**
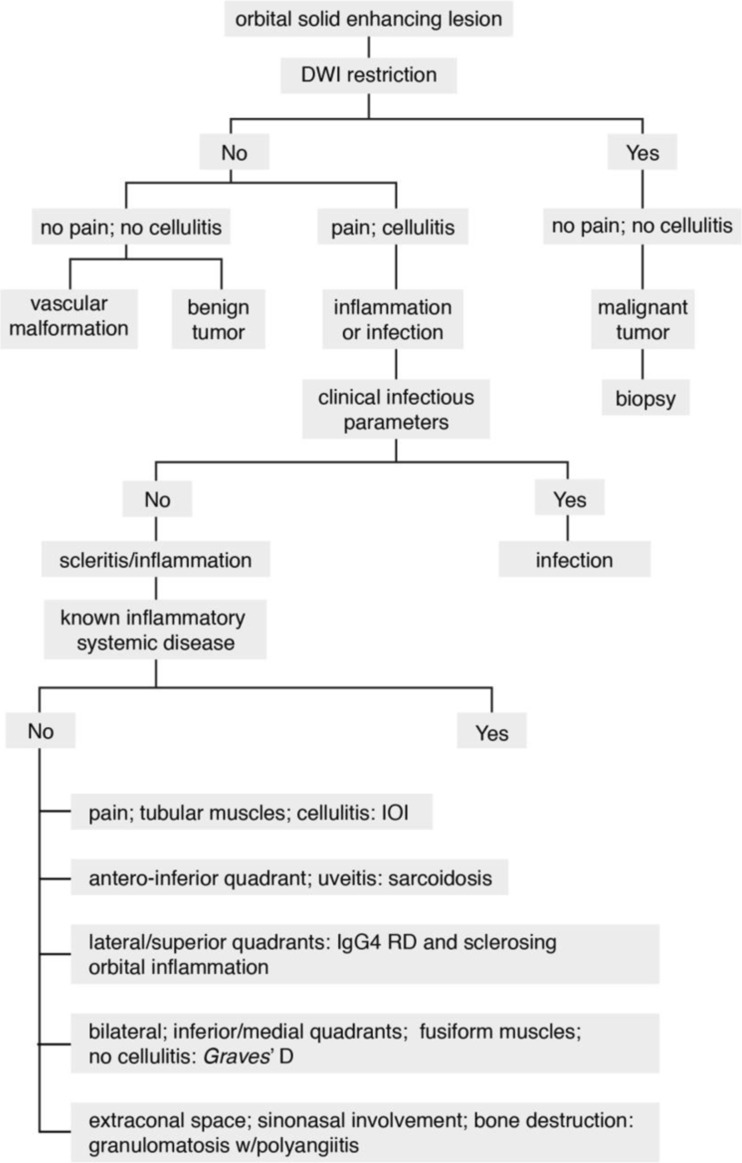
Decision tree in an orbital solid-enhancing lesion. *IOI*, idiopathic orbital inflammation; IgG4 RD immunoglobulin G4-related disease

With those two purposes in mind and with background knowledge of the imaging characteristics of orbital inflammation, we designed a decision tree for an orbital solid enhancing lesion (Fig. 13).

Population is first dichotomized according to the DWI and subsequently whether pain and cellulitis are present. Diffusion restriction with no pain and no cellulitis point to malignant tumor, and biopsy should be envisaged. Facilitated diffusion together with no pain and no cellulitis can still correspond to inflammation, but other diagnosis such as a benign tumor or a vascular malformation should be kept in mind. The absence of diffusion restriction, in the presence of pain and/or cellulitis, favors an inflammatory or infectious process. The differentiation between inflammation and infection is mainly based on clinical features, with long-standing symptoms pointing to inflammation, while the presence of fever, high infectious parameters (v.g. leukocytosis, elevated CRP), and pus, in which case DWI restriction should be expected, suggesting infection. On imaging, the presence of sinusitis and/or an abscess points to infection, while scleritis suggests inflammation.

Imaging may also play an important role on establishing the diagnosis of the underlying inflammatory disease. This is especially important if the patient is not known to harbor any inflammatory systemic disease. In cases the inflammatory process shows predilection for the muscles at the inferior/medial quadrants of the orbits, the involved muscles have a fusiform configuration, and no cellulitis is present, Graves’ disease should be considered. Graves’ disease is mostly bilateral and symmetric and there can be increased intraorbital fat. In cases of IgG4-related disease or sclerosing orbital inflammation, the lateral/superior quadrants of the orbit are preferentially involved and both have an indolent course. IgG4-related disease is mostly bilateral, the involved muscles have a fusiform configuration, and in 30% of the cases, there is enlargement of the infraorbital nerve, which when present is very suggestive of the diagnosis. On sclerosing orbital inflammation, enophthalmus can exist suggesting the fibrotic process. If there is a predilection for the extraconal space, with or without chronic sinonasal involvement, with bone destruction, consider granulomatosis with polyangiitis. History of uveitis and a predilection for the antero/inferior quadrant suggests sarcoidosis. Sarcoidosis is mostly unilateral and with a subacute presentation. When pain is a predominant feature, the involved muscles having a tubular configuration and cellulitis is present, consider idiopathic orbital inflammation. Idiopathic orbital inflammation is mostly unilateral, and when involving the muscles, it affects especially the medial followed by the superior and lateral recti.

The definitive diagnosis of the orbital inflammatory disease is made by combining the radiological pattern with the laboratory findings and characteristics of other organ involvement. The radiological pattern can be specific for a certain type of orbital inflammation such as in Graves’ disease or in granulomatosis with polyangiitis. However, sometimes, these patterns are shared between different etiologies making the imaging pattern not specific. Still the evaluation of the radiological pattern will shorten the differential diagnosis. That is helpful as it can guide the laboratory evaluation and eventual imaging of other organs. When the diagnosis is still unclear, tissue characterization and/or a therapeutical test is needed. An orbital biopsy is easily considered for accessible orbital lesions such as dacryoadenitis. Locations where surgery is difficult or dangerous, such as the orbital apex or around the optic nerve, may confer a higher threshold for biopsy [51, 52].

## Conclusion

Orbital inflammation is frequently mistaken either for orbital infection or malignant tumors, and its underlying cause is often overlooked. Imaging findings obtained through appropriate protocols and knowledge of the most common orbital inflammatory diseases will help shorten the differential diagnosis, with important therapeutic and prognostic consequences. We have therefore combined different imaging and clinical clues that will allow one to recognize an orbital solid-enhancing lesion as inflammatory. Subsequently we have shown how the different radiological patterns will help in differentiate the possible orbital inflammatory diseases. Overall these considerations enable the treating physician to establish an adequate treatment, and at times, a biopsy can be avoided.
